# Aged Microglia in Neurodegenerative Diseases: Microglia Lifespan and Culture Methods

**DOI:** 10.3389/fnagi.2021.766267

**Published:** 2022-01-05

**Authors:** Hyun-Jung Yoo, Min-Soo Kwon

**Affiliations:** ^1^Department of Pharmacology, School of Medicine, Research Institute for Basic Medical Science, CHA University, Cha Bio Complex, Seongnam-si, South Korea; ^2^Research Competency Milestones Program (RECOMP) of School of Medicine, CHA University, Seongnam-si, South Korea

**Keywords:** microglia, aging, neurodegenerative diseases, microglia lifespan, microglia culture

## Abstract

Microglia have been recognized as macrophages of the central nervous system (CNS) that are regarded as a culprit of neuroinflammation in neurodegenerative diseases. Thus, microglia have been considered as a cell that should be suppressed for maintaining a homeostatic CNS environment. However, microglia ontogeny, fate, heterogeneity, and their function in health and disease have been defined better with advances in single-cell and imaging technologies, and how to maintain homeostatic microglial function has become an emerging issue for targeting neurodegenerative diseases. Microglia are long-lived cells of yolk sac origin and have limited repopulating capacity. So, microglial perturbation in their lifespan is associated with not only neurodevelopmental disorders but also neurodegenerative diseases with aging. Considering that microglia are long-lived cells and may lose their functional capacity as they age, we can expect that aged microglia contribute to various neurodegenerative diseases. Thus, understanding microglial development and aging may represent an opportunity for clarifying CNS disease mechanisms and developing novel therapies.

## Introduction

Microglia were recognized as a type of connective tissue or passive bystander of the central nervous system (CNS) physiology for a century since their discovery by Pio del Rio Hortega in 1919. Nowadays, microglia are defined as multifunctional cells that communicate with the peripheral system as well as other CNS cells, such as neurons, astrocytes, and oligodendrocytes, in physiological states. In addition, microglia are not considered as just spectators in CNS pathologies and have been found to play roles as determinants of diseases.

## Microglia Development and Aging

### Microglia Development and Specific New Markers

Microglia are the primary innate immune cells located within CNS parenchyma and have different unique signature genes from other CNS macrophages, such as perivascular macrophage, meningeal macrophage, and choroid plexus macrophage (Li and Barres, 2018). In addition, microglia develop in a stepwise fashion (Matcovitch-Natan et al., 2016), indicating that prenatal and postnatal microglia are different from adult microglia.

**FIGURE 1:**
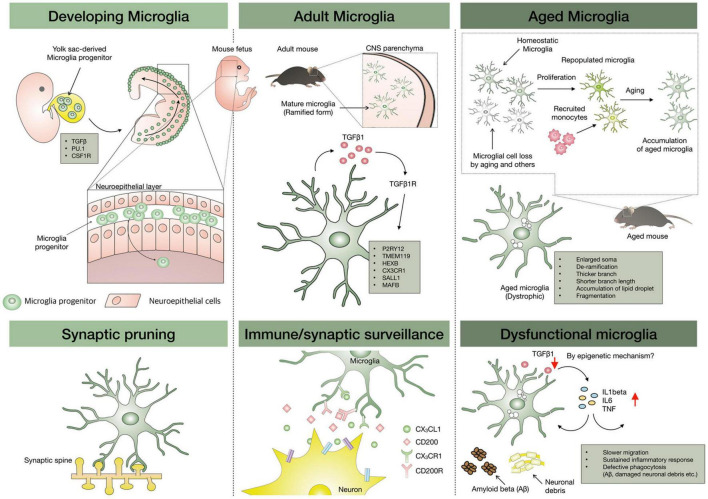
Microglial development, repopulation, and aging. Microglia progenitors are originated from the yolk sac and migrate to the brain parenchyma through the head neuroepithelial layer, and then proliferate and acquire the ramified form with adult microglial signatures. TMEM119, P2RY12, CX3CR1, and HEXB are signature genes on adult microglia, and TGF-β plays a crucial role in their maintenance. With aging, microglial loss can occur and the loss might be replaced by microglial proliferation or infiltrated macrophages distinct from the yolk-sac origin of homeostatic microglia. However, owing to limited repopulating capacity, it is speculated that aged microglia can be accumulated in the aged brain, leading to entire work overload due to relative dysfunctional aged microglia. The epigenetics might be involved in the alteration of homeostatic microglial genes in aged microglia.

One of the reasons microglia can have unique signature genes is associated with their origin and developmental process. Owing to phenotypic similarities to dendritic cells (DCs) and peripheral monocytes/macrophages, the origin of microglia was presumed to be of hematopoietic origin. In fact, many studies have supported this speculation, showing that irradiation-induced myeloablation facilitates infiltration of Ly-6C*^hi^*CCR2^+^ monocytes into CNS (Mildner et al., 2007; Varvel et al., 2012). These data suggest that peripheral monocytes can migrate to CNS parenchyma and settle down with morphological similarities to microglia. However, this occurred only under factitious conditions, and there was no way to confirm whether the engrafted monocytes are truly resident microglia. Meanwhile, Ginhoux et al. (2010) clearly proved that microglia were derived from embryonic yolk sac during development by using *in vivo* fate mapping approach for yolk-sac-derived cells. This observation was confirmed again by Schulz et al. (2012), who showed that Myb^–/–^ mice had a normal number of microglia but were deficient in hematopoietic-derived monocytes/macrophages. Conclusively, microglia are derived from the first wave of hematopoiesis in the yolk sac and not from postnatal hematopoiesis (Ajami et al., 2007). To summarize the overall development of microglia, microglia precursors derived from yolk sac migrate to the brain parenchyma at embryonic day 8.5 (E8.5) in mice (Nayak et al., 2014) and gestational week 12–13 (GW12–13) (Lloyd et al., 2017) in humans and then proliferate and acquire the ramified form through their developmental program, resulting in having their signature genes (developing microglia column in Figure 1). The mature form of microglia contributes to CNS homeostasis by interacting with almost all CNS components as well as the peripheral immune system. In healthy state, microglia dynamically survey the surrounding environment and maintain steady, region-specific densities by self-renewal.

After confirming microglial origin, essential factors for microglia development and maintenance have been suggested in various mutant mice (Li and Barres, 2018). Macrophage colony-stimulating factor (M-CSF or CSF1) is a hematopoietic growth factor produced by endothelial cells, microglia, oligodendrocytes, and astrocytes in CNS and induces differentiation, proliferation, and maturation of macrophages. CSF1 receptor (CSF1R), a receptor tyrosine kinase with two cognate ligands [CSF-1 and interleukin-34 (IL-34)], regulates tissue macrophage homeostasis (Chitu et al., 2016). Recent studies in mice have revealed that CSF1R signaling contributes to the development and maintenance of the microglial population. In CSF1R-mutant mice, yolk-sac macrophages were absent and microglia colonization failed to occur (Oosterhof et al., 2018). Similarly, PLX3397 (CSF1R inhibitor) administration for 7 days eliminates >90% of microglia in adult mice (Elmore et al., 2014). Consistently, microglia-specific *Csf1r* knockout (KO) mice also showed loss of microglia (Buttgereit et al., 2016). Interleukin-34 ablation in neuronal progenitors led to the loss of gray matter microglia in a selective, dose-dependent manner (Badimon et al., 2020). In addition, reports have shown that the development of microglia also relies on the transcription factors interferon regulatory factor 8 (IRF8) and PU.1 (Kierdorf et al., 2013). The cytokine transforming growth factor-β (TGF-β), known as an anti-inflammatory cytokine, is another important factor in the development of microglia and in maintaining the homeostatic function of microglia (Butovsky et al., 2014).

The clarified microglial origin indicates that microglia can have their unique characters distinct from the neuroectodermal origin of other CNS cells. Nevertheless, researchers had no means to detect resident microglia, except for markers such as ionized calcium-binding adaptor molecule (Iba-1), fractalkine receptor [CX3C chemokine receptor 1 (CX3CR1)], and CD11b that are also expressed by CNS-engrafted monocytes/macrophages (Kettenmann et al., 2011). Thus, the absence of microglia-specific markers made it difficult to interpret the role of microglia under the complex neuroinflammatory environment formed by a mixture of microglia and CNS-engrafted monocytes/macrophages. For example, it was almost impossible to distinguish whether core cells of the neuroinflammatory response in multiple sclerosis were resident microglia or engrafted immune cells that permit infiltration of peripheral immune cells into CNS parenchyma. Considering that neuroinflammation is not uniform and has a diverse status, the aforementioned conundrum also applies to other neurological disorders, such as stroke, Alzheimer’s disease (AD), Parkinson’s diseases, amyotrophic lateral sclerosis (ALS), and psychiatric disorders (Rezai-Zadeh et al., 2009; Takahashi et al., 2016; Yang et al., 2020).

The abovementioned concerns have been resolved by the discovery of purinergic receptor P2Y12 (P2RY12) in 2014 (Butovsky et al., 2014) and transmembrane protein 119 (TMEM119) in 2016 (Bennett et al., 2016). Before the discovery of these markers, morphological distinctions, relative marker expression (of the common leukocyte antigen CD45*^hi/lo^*) by flow cytometry, or generating bone marrow (BM) chimeras were used to distinguish microglia from engrafted CNS macrophages and peripheral monocytes (Lassmann et al., 1993; Ford et al., 1995). These techniques presented inherent limitations as these are not unique markers and the chimera model leads to partial chimerism, requiring much effort and time. Meanwhile, with advanced techniques, such as genetics, imaging, mass spectrometry, single-cell technologies, and transcriptome analysis, it has become possible to elucidate the heterogeneity and functional role of microglia in mice and humans. Establishment of CX3CR1CreER mouse lines to target microglia (Goldmann et al., 2013; Yona et al., 2013), identification of a microglia-specific signature using transcriptome analysis (Beutner et al., 2013; Butovsky et al., 2014), identification of microglial heterogeneity and subpopulation depending on brain region (Grabert et al., 2016), sex (Guneykaya et al., 2018; Villa et al., 2018), and neurodegenerative disease using single-cell analysis of murine and human microglia are the examples. Regarding validation of microglia-specific antibody, P2RY12 immunoreactivity (IR) for resident microglia was not co-localized with green fluorescent protein (GFP)-tagged infiltrated monocytes in experimental autoimmune encephalomyelitis (EAE) model with demyelinated pathology by infiltrated immune cells (Butovsky et al., 2014). In mutant superoxide dismutase 1 (mSOD1) mice, P2RY12 IR was rarely detected in the end stage of disease, although many Iba-1-positive cells were co-localized with increased inducible nitric oxide synthase (iNOS) in the spinal cord. Another microglia-specific marker, TMEM119, plays a key role in the validation of the microglial cell model as a signature gene that is expressed only in adult microglia (Bennett et al., 2016). After confirming microglia-specific markers, cell models closer to adult microglia, which can also be known as “microglia-like cells,” have been reported (adult microglia column in Figure 1). This issue is going to be introduced in section “*in vitro* Methods for Aged Microglia Study.” Collectively, these newly derived markers raise the fundamental question about the role of resident microglia during neuroinflammation and disease progression, and this issue is going to be discussed in section “Aged Microglia in Neurodegenerative Diseases.”

### The Origin of Repopulated Microglia

As mentioned above, microglia originate from the yolk sac and not the neuroectoderm. Therefore, we have no choice but to ask the next question. How are microglia replaced if microglial cells are depleted by aging or other stimuli? The possible hypothesis that microglia loss would be supplemented by BM-derived monocytes seems to be reasonable. Acute microglia depletion by pharmacological inhibition *via* CSF1R antagonist induced peripheral monocytes infiltration into CNS without blood-brain barrier (BBB) breakdown, and the infiltrated monocytes showed functional behavior like resident microglia, although transcriptome analysis revealed that the replaced cells did not have the same signature genes as that of resident microglia (Cronk et al., 2018). However, there is a report that acute depletion of microglia could be repopulated by the proliferation of residual microglia rather than *de novo* microglia progenitor differentiation, nestin-positive cells, or peripheral monocytes/macrophages (Huang et al., 2018). However, this study also indicated that transcriptomes of the repopulated microglia were distinct from resident microglia. The debate on the origin of repopulated microglia is still ongoing, but two major studies have a common finding that the repopulated microglia are not the same as yolk-sac-derived resident microglia. This finding is very important since microglia aging may be associated with various neurological disorders (aged microglia column in Figure 1) (Spittau, 2017; Angelova and Brown, 2019). In addition, a heterogeneous population of aged microglia might require a stratified targeting for microglia rejuvenation strategy.

### Microglia Lifespan and Limited Repopulation

Microglia are long-lived cells, and their activities may be dysregulated as they age. In addition, microglia are not replenished by circulating monocytes under homeostatic conditions (Mildner et al., 2007). As mentioned above, microglia can be replenished by repopulation when depleted, but the repopulated microglia are not the same transcriptionally as previous resident microglia. Thus, microglia lifespan is a crucial point in understanding the pathophysiology of neurological disorders. Previously, an indirect study to establish chimerism in circulating BM-derived precursors suggested that microglia lived long in healthy CNS through much of the lifespan of an animal (Mildner et al., 2007). More recently, by *in vivo* single-cell imaging, it was found that the median lifetime of neocortical-resident microglia was over 15 months, and approximately half of total microglia survived the entire mouse lifespan, suggesting that microglia are long-lived cells and microglial replenishment may be less required relatively than other CNS cells (Fuger et al., 2017). In humans, an indirect method referring to the ^14^C atmospheric curve was used to analyze the lifespan and turnover of microglia. Human microglia renewed at a median rate of 28% per year and the average lifespan was 4.2 years. Most of the microglia population (96%) was renewed throughout life, suggesting that the microglia population in the human brain is maintained by persistent slow turnover throughout adult life (Reu et al., 2017). Thus, the persistence of individual microglia throughout life explains how microglial aging may contribute to age-related neurodegenerative diseases.

Is endless repopulation of adult microglia possible? Adult microglia can be depleted by 90% by CSF1R inhibitor in mice. Once microglia are depleted acutely, withdrawal of inhibitor promotes repopulation of microglia in the entire CNS, and greater depletion of microglia results in more rapid repopulation (Najafi et al., 2018). Interestingly, this study found that the recovery time was gradually extended as the depletion was repeated, indicating the possible limited capacity for microglial repopulation. Thus, maintaining yolk-sac-derived microglia in a healthy state for a long time can be a good strategy to prevent age-related neurodegenerative diseases.

### Aged Microglia in Neurodegenerative Diseases

Aging is associated with altered inflammatory status in the brain as well as systemically. As CNS ages, microglial morphology and number also change. Aged microglia in humans demonstrate dystrophic morphologies, indicating fragmentation of residual processes, less branching, deramified dendritic arbors, and cytoplasmic beading in shape relative to young microglia depending on the observed region (Streit et al., 2004). The dystrophic microglia are contrasted morphologically and functionally with dark microglia showing the condensation of their cytoplasm and nucleoplasm, accompanied by cytoplasm shrinkage, Golgi apparatus, and endoplasmic reticulum dilation, highly ramified morphology, and increased phagocytosis (Bisht et al., 2016). Along with this morphological change, homeostatic microglial functions decline with aging. Homeostasis is defined as a relative constancy of set point formed in certain conditions, and maintaining homeostatic microglial function indicates an effort to restore the deviating set point due to aging in the CNS environment (Deczkowska et al., 2018). The homeostatic microglial function indicates timely proper response required at each stage of life. Because excessive or tolerant microglial response can interrupt the tissue restoration after CNS damage, the transition from homeostatic microglial function in steady-state to immune-modulating mode under pathological conditions should be tightly regulated. Hence, microglial immune checkpoints, which are a set of controlling mechanisms preventing uncontrolled response in microglia, were suggested (Deczkowska et al., 2018). CX3CR1, also known as the fractalkine receptor, is a transmembrane protein and chemokine for leukocyte migration expressed on monocytes, DCs, and microglia (Harrison et al., 1998). CX3CL1, a ligand of CX3CR1, is expressed on the neuronal surface or released as the active soluble form from specific neurons. Tight regulation between neuronal CX3CL1 and microglial CX3CR1 controls microglial functional phenotype and their hyperactivation under an inflammatory environment. For example, CX3CR1 deficiency in mice with an induced exaggerated response to lipopolysaccharide (LPS) stimuli in CNS showed microglial neurotoxicity and advanced neuronal death (Cardona et al., 2006b). CD200 receptor (CD200R) in microglia also interacts with neighboring cells, including neurons, astrocytes, oligodendrocytes, and endothelial cells through their CD200 ligand; this has also been suggested as a mechanism of attenuating microglial activation, primarily under inflammatory conditions (Walker and Lue, 2013).

Another homeostatic transcriptional regulator of microglia is TGF-β produced by astrocytes and microglia at high levels in healthy CNS (Butovsky and Weiner, 2018). TGF-β KO microglia show aberrant immune-activated signature, increased neuronal death, reduced synaptic plasticity, and late-onset motor deficits (Brionne et al., 2003). Transcription factors (MafB, Mef2C, and Sall1) and MeCP2 as a methylated DNA binding repressor are also involved in controlling microglial immune activity. Congenital disruption of the *MafB* gene in microglia induced enhanced inflammation in adult mice (Matcovitch-Natan et al., 2016). Mef2C, which is expressed in microglia, limited microglial immune activation in response to pro-inflammatory perturbations (Potthoff and Olson, 2007). MeCP2 also aggravates immune response to tumor necrosis factor (TNF) (Cronk et al., 2015). Sall1, which controls the transcriptional signature of microglia, regulates microglial identity and physiological features in the CNS (Buttgereit et al., 2016). In this study, *Sal1* deficiency in microglia induced their activation and disturbed adult hippocampal neurogenesis.

As described above, changes in several immune checkpoints can affect microglial homeostatic function that is orchestrated by checkpoint mechanisms throughout life. Interestingly, microglial immune checkpoints are distorted with aging, indicating dysregulation of homeostatic microglial function (Deczkowska et al., 2018). Aged microglia display increased immune vigilance (high expression of both immunoreceptors and an inflammatory secretome) along with dysregulated phagocytosis (Grabert et al., 2016). The increased release of neurotoxic substances and reduced ability to phagocytose debris and toxic protein aggregates in dystrophic microglia leaves neurons vulnerable. Insufficient phagocytic activity of aged microglia toward apoptotic bodies, misfolded protein aggregates, and myelin might result in the gradual accumulation of potentially toxic compounds, a hallmark of age-related neurodegenerative diseases (Safaiyan et al., 2016; Galloway et al., 2019; Damisah et al., 2020). The cause of such phenotypic shift in aged microglia appears to be related with changes in the microglial homeostatic gene profile. In essence, directly isolated microglia from aged human brain also support the observation that aged human microglia exhibit downregulated TGF-β signaling in Kyoto Encyclopedia of Genes and Genomes (KEGG) pathway (Olah et al., 2018). This report suggests that diminishing TGF-β signaling highlights the perturbation of homeostatic programs as microglia activate reactive pathways to respond to aging-related changes such as the accumulation of amyloid pathology. Another study using cortical microglia purified from postmortem human samples clearly demonstrated aged microglia-associated gene profiles such as cell surface receptor P2RY12 and cell adhesion molecules (Galatro et al., 2017). Microglial functional phenotype can be regulated by TGF-β produced by astrocytes and neurons among other cells (von Bernhardi and Ramirez, 2001; Chen et al., 2002; Herrera-Molina and von Bernhardi, 2005). TGF-β promotes phagocytosis and neuronal protection, depending on the Smad3-mediated mechanism in microglia (Tichauer et al., 2014; von Bernhardi et al., 2015). Thus, aging or loss of the TGF-β releasing cells might affect microglial TGF-β signaling and their homeostatic genes expression. Changes in gene expression in aged microglia may also be based on changed epigenetics. Microglia plasticity can be controlled by epigenetics (Cheray and Joseph, 2018), and aged microglia show upregulation of *IL-1*β gene expression by hypomethylation of CpGs sites on *IL-1*β proximal promoter (Matt et al., 2016). Similarly, a unique epigenome and transcriptome can define a phenotype of microglia in aging, including changes in homeostatic microglial genes such as *TGF*-β, *CX3CR1*, and *P2RY12*.

Along with extreme longevity (Fuger et al., 2017) and limited repopulation capacity (Najafi et al., 2018) of microglia, turnover of aged microglia does not reset the pro-inflammatory phenotype in the aged CNS microenvironment (O’Neil et al., 2018). In addition, the homeostatic microglia population gradually decreases with aging (Niraula et al., 2017), leading to work overload for the remaining microglia. Thus, an intrinsic dysfunction of aged microglia is closely related to neurodegenerative disease. “Dystrophic microglia” refers to microglial morphological changes with age (Streit and Xue, 2014); they have been detected in the periphery of tau and amyloid pathology in the brains of patients with AD and likewise near sites of Lewy bodies in the brain of patients with dementia with Lewy bodies (Streit and Xue, 2016; Shahidehpour et al., 2021). In particular, microglia activation occurs at the early stages of AD, and as it disappears, microglia become senescent/dystrophic and less responsive to stimuli at a late stage (Graeber and Streit, 2010). Histopathological finding from 19 AD pathologies indicates that aging-related microglial degeneration rather than microglial activation might contribute to the onset of AD (Streit et al., 2009). In fact, aged microglia-related releasing factors disturbed clearance of apoptotic bodies and aggregation of α-synuclein, thus, aggravating disease progression (Angelova and Brown, 2019). In mSOD1 mice, which is a familial ALS mouse model, microglia were involved in inflammatory reactions in the early stage, and they exhibited a tolerant and dystrophic form that does not function properly at the end stage of disease progression, demonstrating P2RY12 IR disappearance, despite Iba-1 IR increase in the spinal cord (Butovsky et al., 2015). Similarly, acutely isolated mSOD1 microglia in the symptomatic period showed β-galactosidase activity as well as the elevation of p16, matrix metalloproteinase-1 (MMP-1), p53, and nitrotyrosine with large and flat morphology, suggesting a senescence-associated secretory phenotype (SASP) (Trias et al., 2019). Chronic amyloid β exposure induced microglial impairment with immune tolerance, which was associated with microglial metabolic defects (downregulation of mTOR-glycolysis pathways) (Baik et al., 2019). Chronic stress, which is an aggravating factor in AD and risk factor in mood disorders, also sensitized microglia toward a primed phenotype in the acute stage, and, subsequently, led to dystrophic morphology according to stress duration in mice, suggesting that chronic depression may be associated with dystrophic microglia (Kreisel et al., 2014).

At this point, one question is why multiple studies thus far have suggested that inflammatory activation of microglia is the main culprit of neurodegenerative diseases, although microglia have dystrophic morphology and lose their homeostatic genes. One of the main causes is associated with previous microglial markers such as Iba-1 and CD11b that cannot discriminate resident microglia from infiltrated monocytes/macrophages because microglia signature genes, including P2RY12 and TMEM119, were established after 2014 as mentioned above. Thus, papers published before 2014 seem to have mistaken the main culprit for the neuroinflammatory response as resident microglia rather than Iba-1- (or other previous markers) positive cells, detecting both infiltrated monocyte/macrophages and resident microglia in neurodegenerative diseases with BBB breakdown. Another possible reason is related to immature features of fetal or neonatal microglial cells, widely used as *in vitro* surrogates. Single-cell analysis according to developmental state clearly identified that fetal/neonatal microglial cells had a different signature from acute-isolated adult microglia in mice (Matcovitch-Natan et al., 2016), and microglial cell lines, as well as fetal/neonatal microglial cells, rarely express adult microglial signature genes (Butovsky et al., 2014). Regarding microglial functional character, acute-isolated microglia from post-mortem human brain tissue showed a tightly regulated phenotypic change to an inflammatory environment composed of LPS and interferon-γ (IFN-γ) compared with neonatal/fetal microglia (Melief et al., 2012). Thus, we cannot exclude the possibility that immature microglial cells present more dynamic inflammatory reactions to inflammatory stimuli distortions than actual adult microglia. Based on these reports, it may be concluded that the responsibility for neuroinflammation in neurodegenerative diseases cannot be shifted only to the yolk-sac origin of homeostatic microglia rather than infiltrated monocyte/macrophages because dystrophic and tolerant microglia were also observed in most neurodegenerative diseases (Streit et al., 2009; Varvel et al., 2016; Karlen et al., 2018; Sevenich, 2018).

## Functional Change in Aged Microglia

The change in microglial transcriptome with aging, microglial functions, such as phagocytosis, synaptic pruning, migration, and cytokine release to stimuli, can be also altered toward a decline or more dysregulation in the supportive and protective capacity. Microglia are remarkably versatile in their functions that overall achieve a homeostatic environment. Microglial dysfunction has been linked to neurodegenerative diseases. Live imaging of retinal microglia in young and aged mice revealed that aged microglia showed slower process motilities in homeostatic state and slower migrating response to laser-induced focal tissue damage (Damani et al., 2011). In addition, aged retinal microglia exhibited a sustained inflammatory response and defective phagocytosis (Damani et al., 2011). Aged microglia exhibited a heightened and prolonged response to inflammatory stimuli and showed a blunted response to IL-4, suggesting a reduced repair mechanism (Fenn et al., 2014). Furthermore, defective phagocytosis of myelin debris by aged microglia led to impaired remyelination (Rawji et al., 2020). *Ex vivo* cultured microglia isolated from the brain of aged mice constitutively secreted more amounts of pro-inflammatory cytokines, such as TNF-α and IL-6, and exhibited less Aβ phagocytosis, leading to higher amyloid burden (Njie et al., 2012). Proteomic analysis of aged microglia isolated by CD11b magnetic beads showed that aged microglia exhibited disruption in chromatin remodeling, loss of nuclear architecture, and impairment in RNA processing (Flowers et al., 2017). In this study, aged microglia showed a bioenergetic shift from glucose to fatty acid utilization, linking with the study results that restoration of defective glycolytic metabolism could be a target for boosting the tolerant microglia induced by chronic amyloid β exposure (Baik et al., 2019). A recent study demonstrated that aged microglia were not uniform throughout the brain but had transcriptomic diversity in a region-dependent manner, indicating differential susceptibility to aging factors (Grabert et al., 2016).

Considering that microglial phagocytic function contributes to clearance of aberrant proteins (amyloid β, Apolipoprotein E, and α-synuclein), and damaged neuronal debris, synaptic stripping, and remodeling for CNS homeostasis (Wake et al., 2013), decrease in phagocytic function with aging potentially have a direct link with increased susceptibility to the progression of neurodegenerative diseases. Signaling between CX3CL1 and its receptor CX3CR1 is critical for microglial synaptic pruning, phagocytosis, and migration in the adult brain; however, in the aged brain, their expression levels are decreased (Wynne et al., 2010; Deczkowska et al., 2018). In contrast, hallmarks of microglia activation such as major histocompatibility complex II (MHC II) and CD86, Toll-like receptors (TLRs), and nucleotide oligomerization domain (NOD)-like receptors (NLRs) are increased with age (Patterson, 2015). Age-dependent microglia dysfunction might be enhanced by the loss of endogenous TGF-β1 to maintain mitochondria homeostasis. TGF-β1 induces microglia phagocytosis of apoptotic cells *via* Mfge8 expression (Spittau et al., 2015).

Microglia priming is a stronger response than that of the stimulus-naïve microglia to a second inflammatory stimulus (Perry and Holmes, 2014). The exaggerated response to toxic stimuli, such as LPS, has been considered as a “primed state” of microglia with overproduction of pro-inflammatory cytokines or decreased anti-inflammatory factors. The “priming state” indicates a phenotypic shift of microglial cells toward a more sensitized state, responding to an additional stimulus more rapidly, longer, and to a greater degree than expected if non-primed (Harry, 2013). This exaggerated inflammatory response can compromise critical processes for optimal cognitive functioning. For example, IL-1β production in aged brain interrupted hippocampus-dependent memory systems and synaptic plasticity processes *via* disruption of brain-derived neurotrophic factor (BDNF) function (Norden and Godbout, 2013; Patterson, 2015). In addition, when aged mice received an intraperitoneal injection of LPS or *Escherichia coli*, IL-1β production was significantly higher and for a longer time than that in young mice (Godbout et al., 2005; Barrientos et al., 2009).

In summary, aged microglia are in a primed state and show an exaggerated response to inflammatory stimuli. In addition, aged microglia respond slowly to toxic stimuli, lose dynamic surveillance features, and exhibit reduced phagocytic function. These results were derived by live imaging using CX3CR1-specific GFP-tagged microglia; tissue staining using microglial markers, such as Iba-1 or CD11b, in aged mice; and acutely isolated microglia using CD11b magnetic beads or Percoll gradient. However, these methods cannot isolate perfectly pure resident microglia distinct from infiltrated monocyte/macrophage or CNS macrophages located in choroid plexus, meninges, and perivascular space, and we cannot determine the contaminated amount. In addition, an advanced dynamic contrast-enhanced magnetic resonance imaging protocol with high spatial and temporal resolutions quantified regional BBB permeability in the living human brain and found an age-dependent BBB breakdown in CA1 and dentate gyrus subdivisions of the hippocampus, supporting infiltration of macrophages and monocytes into CNS parenchyma (Montagne et al., 2015). Furthermore, we cannot discriminate whether higher inflammation to peripheral LPS injection is due to aged microglia or infiltrated macrophages/monocytes because peripheral LPS injection induces BBB disruption (Banks et al., 2015). Actually, highly pure microglia (CD11b*^high^*CD45*^int^*) isolated from the human parietal cortex with the elimination of meningeal macrophages by fluorescence-activated cell sorting (FACS) indicated that microglia of physiologically aged mice do not recapitulate the effect of aging on human microglia, and the top 100 differentially expressed genes in human aged microglia were more related with actin cytoskeleton-associated genes, sensome cell surface receptor, cell adhesion molecules, and surface receptors rather than inflammatory cytokines. These results suggest that decline in fine microglial processes, such as motility for surveillance, perturbed microglial migration, and reduced phagocytosis efficiency, may be associated with age-related neurodegeneration (Galatro et al., 2017). In a mouse model of telomere shortening (mTerc^–/–^), it seems that peripheral LPS injection enhanced pro-inflammatory response in mTerc (^–/–^) microglia, but the enhanced inflammatory response was not accompanied with genes related with aged microglia and correlated closely with infiltration of immune cells (Raj et al., 2015). Thus, the primed state of aged microglia might need to be reevaluated with purer isolated microglia with a stably expressed core marker during homeostasis and disease, especially according to neurodegenerative disease progression.

### *In vitro* Methods to Study Aged Microglia

**FIGURE 2:**
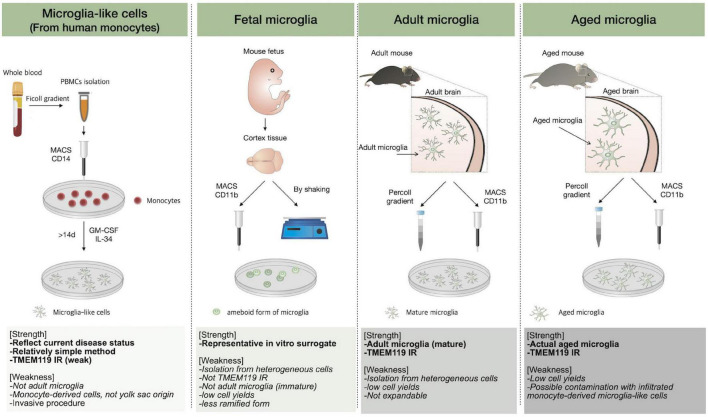
*In vitro* microglial culture. The methods to obtain microglial cells are described and the strengths of each technique, followed by weaknesses, are listed.

Microglia are widely involved in the homeostatic maintenance in the CNS, and age-associated microglial dysfunction is closely related to CNS diseases. Proper use of *in vitro* methods recapitulating adult microglia is required to study microglia; however, it has been difficult to recapitulate adult microglial cells perfectly due to the complexity of the origin and developmental process. In this section, we introduce the currently used *in vitro* methods for an accurate understanding of microglia. The features and limitations of each method are discussed, briefly referring to well-organized previous review papers (Timmerman et al., 2018; Angelova and Brown, 2019). A brief description of *in vitro* methods is illustrated in Figure 2. The description of “aged” microglia may indicate the inclusion of several distinct phenotypes and the term is uncertain (Koellhoffer et al., 2017). Based on the fact that senescent microglia or aged-like phenotype is not sufficient to cover aged microglia features, we have tried to use the terms as distinctly as possible. SASP as an alternative method to characterize aged microglia was indicated separately (Streit and Xue, 2014). Actually, aged microglia seem to have distinct features from *in vitro* senescent microglia although both aged microglia and senescent microglia show dysfunctional phenotypes such as impaired phagocytosis, slow migration, slow response to stimuli. Thus, *in vitro* senescent microglia might not recapitulate aged microglia perfectly so far.

### Microglial Cell Lines

Initially, a cell line was suggested as a solution to the problem of not being able to secure enough microglial cells for detailed studies (Blasi et al., 1990; Nagai et al., 2001). The microglial cell line was established by immortalization. Such methods include viral transduction with oncogenes (e.g., v-raf, v-myc, v-mil), SV 40 T antigen, and cancerization (e.g., p53-deficient cell) using cells derived from various species, including mouse, rat, macaque, and human (Timmerman et al., 2018). Infinite growth capacity due to immortalization enables passage culture, and it is useful in research methods such as high-throughput screening assays that require a high number of cells, due to their relatively high growth rate (Dello Russo et al., 2018). However, immortalization is a double-edged sword. Immortalization has the advantage of being able to easily obtain a tremendous number of cells but rather distorts the properties of microglia due to artificial manipulation. Thus, they are different from adult microglia in genetic and functional aspects (Butovsky et al., 2014; Das et al., 2016; Melief et al., 2016). Immortalized cells may also be not suitable for studying long-lived adult microglial cells, which show very low proliferative capacity at a healthy state (Fuger et al., 2017; Haenseler et al., 2017). For studying microglia senescence, there is a report that repeated LPS stimulation (10 ng/ml, every 48 h) can induce cellular senescence in BV2 cells (Yu et al., 2012). In this study, BV2 senescence was evaluated by β-galactosidase staining, p53, and cell cycle arrest in G0/G1 phage, suggesting that multiple inflammatory stimuli may induce microglia senescence.

### Primary Fetal/Neonatal Microglial Culture

Rodent primary microglia are commonly obtained from neonatal/fetal animals (Giulian and Baker, 1986), and human primary microglia also may be obtained from embryonic or fetal tissues (Satoh and Kim, 1994). After tissue collection, it is necessary to isolate a sufficient amount of only the desired microglial sample of high purity. There are several enzymatic and mechanical separation methods. In this process, one method is density gradient centrifugation; this method can isolate microglia with more than 99% purity (Cardona et al., 2006a; Zuiderwijk-Sick et al., 2007). Other ways are magnetic-activated cell sorting (MACS) (Nikodemova and Watters, 2012; Mizee et al., 2017) and FACS (Olah et al., 2012; Bennett et al., 2016); these methods use microglia antibody coated with magnetic beads and fluorescent material, respectively, and the other is to perform the shaking procedure (Tamashiro et al., 2012).

Rodent primary microglia alone are insufficient to study human microglia due to interspecies differences with regard to features of adhesion, proliferation rates, and expression of key receptors (Smith and Dragunow, 2014). Because no artificial treatment, such as genetic modification, is applied, fetal/neonatal microglia culture has the advantage that it is similar to resident microglia compared with cell lines, but passage culture is difficult, and too many animals are required to obtain a high number of cells. Notably, early fetal or neonatal microglia differ in many ways, including transcriptome, function, morphology, and physiology, from adult microglia settled in the adult brain after BBB formation (Butovsky et al., 2014; Matcovitch-Natan et al., 2016; Prinz et al., 2019). For induction of senescent microglia, long-term culture using fetal microglia was proposed (Caldeira et al., 2014). In this study, 16 days *in vitro* (16 DIV) cultured microglia showed slightly increased ramified morphology compared with the ameboid form of 2 DIV and showed reduced migration and phagocytosis compared with 2 DIV. In addition, 16 DIV exhibited enhanced β-galactosidase staining and decreased autophagy, indicating that this method induces senescence in microglia. HIV-1 also induces a senescence-like phenotype in human microglia (Chen et al., 2018). Primary human fetal microglia exposed to single-round infectious HIV-1 pseudotypes had significantly elevated senescence-associated β-galactosidase activity, p21 levels, and production of cytokines (such as IL-6 and IL-8), which are potentially indicative of a SASP, and showed mitochondrial dysfunction. Another method to induce β-galactosidase activity in microglia is dexamethasone (DEX) treatment (Park et al., 2019). In this method, we found that DEX induced ramified form but showed dysfunctional phagocytosis and tolerant response (decreased mRNA of pro- and anti-inflammatory cytokines) with downregulated homeostatic genes, such as *Cx3cr1*, *Cd200r*, *P2ry12*, and *Trem2*. They were partially unlike aged microglia because DEX-treated microglia showed increased autophagy and decreased inflammatory cytokines. Based on the fact that dystrophic microglia can be identified by high ferritin, iron content also can generate a microglia model of an aged-like phenotype (Brown, 2009).

### Direct Isolation of Adult Microglia and *ex vivo* Microglial Culture

As the need for adult microglia has emerged, many studies have directly isolated microglia from adult animals. This is performed in a similar way to primary fetal/neonatal microglia culture, with the difference that adult microglia can be obtained after mechanical and enzymatic dissociation of the rodent brain. Generally, after digestion, adult microglia are separated from the cocktail containing collagenase and dispase using a discontinuous Percoll gradient or MACS or FACS (Becher and Antel, 1996; Cardona et al., 2006a; Nikodemova and Watters, 2012; Olah et al., 2012; Bennett et al., 2016; Mizee et al., 2017). Microglia obtained in this way can be used for (single-cell) transcriptome analysis, high-density microarray, proteomic, or cytometric analysis. The main advantage of this method is the ability to obtain adult microglia with specific features as mentioned above. Namely, it can reflect the state of microglia present in the adult brain environment, although acutely isolated adult microglia rapidly lost TMEM119 expression in culture media (Bohlen et al., 2017). Although it has limitations like inefficient passage culture and low cell yield leading to a high number of animals being used (Timmerman et al., 2018), it is undeniably the most reliable way to obtain actual aged microglia from aged rodents or humans. Notably, the actual feature of aged microglia was confirmed by these methods (Galatro et al., 2017; Olah et al., 2018; Ximerakis et al., 2019). However, direct isolation from aged animals for experiments requires high effort and can be time-consuming. Alternatively, *Ercc1* mutant mice, a DNA repair-deficient mouse that exhibits characteristics of accelerated aging in CNS and other tissues, might be used for studying aged microglia without long aging periods, but this may not reflect natural aging due to genetic manipulation (Raj et al., 2014). Acutely isolated murine microglia from aged mice show different features from senescent microglia obtained by long-term fetal microglia. Senescent microglia exhibited shortened telomeres with increased telomerase activity, whereas aged microglia showed unaltered telomeres and reduced telomerase activity (Stojiljkovic et al., 2019). In this study, senescent microglia showed increased p16, p21, and p53 expression, while aged microglia only exhibited p16 elevation, suggesting that aged microglia show dysfunctional features but cannot exhibit key senescence markers.

### Microglia-Like Cells From Human Induced Pluripotent Stem Cells (iPSCs)

Extracting living brain cells containing microglia directly from the animal or human brain presents technical and/or ethical problems. To overcome these issues, *in vitro* models mentioned below have been proposed. iPSCs or monocyte-derived microglia-like cells were established along with new adult microglial markers.

Induced pluripotent stem cells are reprogrammed adult cells, such as fibroblasts, generated by introducing four transcription factors (Oct3/4, Sox2, c-Myc, and Klf4) (Takahashi and Yamanaka, 2006). The existing *in vitro* microglia models, such as primary cultures, present difficulty in obtaining sufficient normal and disease-associated microglial cell sources. In addition, microglia are very sensitive to the environment in their identity, so they quickly lose their characteristics when separated from the brain microenvironment (Butovsky et al., 2014; Bohlen et al., 2017). To resolve these limitations and reflect microglial development in the *in vitro* model as much as possible, many studies have been conducted on the protocols for *in vitro* differentiation of iPSCs into microglia-like cells.

For the first time, a robust protocol for differentiating human iPSCs and embryonic stem cells (ESCs) into microglia-like cells using the embryonic body (EB) was suggested (Muffat et al., 2016). The microglia-like cells were cultured in serum-free conditioned media to reflect the development environment of actual microglia. These microglia-like cells show characteristics of human primary fetal and adult microglia in gene expression, signature marker, and microglial function (e.g., phagocytosis). In addition, they particularly expressed the markers P2RY12 and TMEM119 and progressively showed a ramified form. Unlike most other iPSC-derived microglia protocol results, this approach showed that iPSC-derived microglia-like cells have features of adult microglia as well as human primary fetal microglia.

Another protocol differentiated iPSCs into human microglia-like cells through exposure to defined factors following the astrocyte co-culture protocol, which includes factors involved in proliferation, such as IL-3, M-CSF, and granulocyte–macrophage-CSF (GM-CSF), in the medium (Pandya et al., 2017). Before final differentiation into microglia-like cells, first, an intermediate process of differentiation into hematopoietic progenitor-like cells (iPSC-HPC) is performed. This makes it possible to analogously reflect the ontogeny procedure of microglia. iPSC-HPC has marked expression of CD34 and CD43, markers of hematopoietic cells. Subsequently, as the differentiation into human microglia-like cells progresses, more microglia-related markers, such as CD11b, Iba-1, HLA-KR, TREM2, and CX3CR1, were expressed.

In a similar vein to the study of Muffat et al. (2016), Abud et al. (2017) published a fully defined serum-free protocol that ensures high purity (>97%) and large quantities. Initially, CD43^+^ iPSCs differentiate into myeloid progenitors by exposure to defined medium and transient low oxygen levels (5%). After 10 days, the medium is replaced with serum-free media containing M-CSF, IL-34, TGF-β1, and insulin. Thereafter, microglia-like cells are exposed to CD200 and CX3CL1 and continue to mature, showing more ramified forms as they mature. Gene expression analysis and functional assessment demonstrated that these microglia-like cells highly resemble human fetal and adult primary microglia. Furthermore, Abud et al. (2017) demonstrated the effect of co-culture with other neural cells on morphology and function as well as gene expression in microglia.

Based on microglial origin, Douvaras et al. (2017) described a reproducible protocol that uses PSC-derived myeloid progenitors, which are considered to correspond to *in vivo* primitive yolk-sac myeloid progenitors in chemically defined conditions. PSCs, including ESCs and iPSCs, are stimulated with a myeloid inductive medium and treated with microglia-promoting cytokines. As a result, KDR^+^ CD235a^+^ primitive hemangioblasts are generated, which then change to CD45^+^ CX3CR1^+^ microglial progenitors *in vitro*. Subsequently, the addition of IL-34 and GM-CSF to plated microglial progenitors differentiates into iPSC-derived microglia-like cells (Ohgidani et al., 2014), ramifying with highly motile processes and monitoring the microenvironment like *in vivo* microglia (Davalos et al., 2005). iPSC-derived microglia-like cells express not only typical microglial markers, such as IBA1, CD11c, TMEM119, P2RY12, CD11b, and CX3CR1, but also signature genes in human primary microglia, such as C1QA, GAS6, GPR34, MERTK, P2RY12, and PROS1 (Butovsky et al., 2014). Furthermore, they showed phagocytosis and intracellular Ca_2_^+^ transient in response to ADP.

To recapitulate the ontogeny of microglia, Haenseler et al. (2017) established a very efficient human iPSC-derived microglia model analogous to the microglial ontogenetic development process. Microglia originate from yolk-sac-derived macrophages, which have *MYB*-independent and *PU.1*- and *Irf8*-dependent properties (Schulz et al., 2012; Kierdorf et al., 2013). To consider this fact, embryonic MYB-independent iPSC-derived macrophages, which were harvested from EB cultured with BMP4, VEGF, SCF, IL-3, and M-CSF, were co-cultured with iPSC-derived cortical neurons for 2 weeks. The obtained iPSC-derived microglia-like cells express major microglia-specific markers, form highly dynamic ramified morphology, and perform phagocytosis. In addition, transcriptome analysis results are similar to those of human fetal primary microglia. In particular, the resulting co-cultures upregulate homeostasis-related function pathways, downregulate pathogen response pathways, and promote enhanced anti-inflammatory response compared with corresponding monocultures. This protocol avoids repetitive cell sorting or replating, resulting in relative simplicity, high efficiency, and yield.

Above mentioned iPSC-derived microglia-like cells have the advantage of securing a sufficient cell source. Another advantage is that iPSC-derived cells of normal donors can be compared with those from patients with neurological disorders, and the genetic background of the patient can be considered. However, despite these advantages, research using iPSC-derived microglia-like cells has limitations to overcome. First, there are too many models with different protocols. To describe the most reliable approaches, comparison and integration between each approach are necessary (Timmerman et al., 2018). In addition, most microglia *in vitro* models using iPSC are inefficient due to low yields, over-time, and cost (Li and Barres, 2018). The effects of the CNS microenvironment cannot be reflected, and most of the iPSC-derived microglia-like cells studied so far have characteristics of primary microglia and not adult microglia. Therefore, developing methods for differentiating microglia-like cells similar to adult microglia may be better suited for neurodegenerative disease studies. Above all, because iPSC technology accompanies cell rejuvenation, iPSC cannot reflect the aging feature of the origin cells obtained from aged humans (Mertens et al., 2015). To our best knowledge, there is no report on *in vitro* aging method utilizing microglia-like cells derived from iPSCs yet.

### Monocyte-Derived Microglia-Like Cells

There are other methods to obtain microglia-like cells by using monocytes. iPSCs-derived microglia-like cells have limitations that they do not reflect the current physiological and pathological status due to rejuvenation (Mertens et al., 2015), but monocyte-derived microglia-like cells have the advantages that they mirror the state of the donor (Ohgidani et al., 2015; Ryan et al., 2017; Sellgren et al., 2017). Thus, induced microglia-like cells from monocytes of aged humans may reflect aged microglia. However, this needs to be validated.

Previously, it has been proven that rat monocytes or macrophages cultured in an astrocyte-conditioned medium (ACM) develop into microglia-like cells showing characteristics of microglia, including ramified morphology (Kettenmann and Ilschner, 1993; Schmidtmayer et al., 1994). Based on this, Leone et al. (2006) showed that human monocytes cultured in ACM exhibit microglia-like features in many respects. Later, it was found that GM-CSF and IL-34, cytokines secreted by astrocytes (Guillemin et al., 1996; Noto et al., 2014), are at least essential in inducing microglia-like cells from human peripheral blood cells (Ohgidani et al., 2014). In particular, IL-34 was found to be a major factor in the proliferation of microglia (Gomez-Nicola et al., 2013). Within 2 weeks, using a cocktail of GM-CSF and IL-34 successfully induced microglia-like cells from human monocytes. These cells represented the various characteristics of microglia, including ramified morphology; markers, such as high CD11 and CX3CR1 and low CD45 and CCR2; phagocytosis; and releasing cytokines related to inflammation (Ohgidani et al., 2014). In the following studies, these induced microglia-like cells were demonstrated to enable various approaches to study microglia in psychiatric disorders by translational research. This can be linked with drug efficacy screening and personalized medicine to maximize the therapeutic effect (Ohgidani et al., 2015). Gene expression analysis showed that microglia-specific genes involved in microglial function are also expressed in monocyte-derived microglia (Ryan et al., 2017). Representative microglial genes TGFBR1 and C1QB are important mediators for synaptic pruning (Bialas and Stevens, 2013), PROS1 is involved in phagocytosis (Fourgeaud et al., 2016), and P2RX7 induces activation and proliferation of microglia (Monif et al., 2009). In addition, monocyte-derived microglia-like cells have also been applied to translational research (Ohgidani et al., 2015; Ryan et al., 2017; Sellgren et al., 2017).

However, as the progenitor of microglia is early erythroid myeloid progenitors (eEMP) originating from hematopoietic stem cells, there is a fundamental limitation that monocyte-derived microglia-like cells and microglia differ in their origins. Moreover, obtaining enough microglia-like cells from human blood monocytes requires repeated invasive procedures (Beutner et al., 2013).

Taken together, each method for culturing microglia *in vitro* has advantages and disadvantages. Although monocyte-derived microglia-like cells can better recapitulate aging as they are not rejuvenated during reprogramming as are iPSCs-derived microglia-like cells, it has not been confirmed whether monocytes obtained from aged humans can actually differentiate to microglia-like cells from aged humans and reflect their features. In the case of rodent microglia, several methods for inducing senescence have been proposed as mentioned above, but the *in vitro* methods require a significant amount of primary microglial cells. To address this issue, our laboratory developed a system to obtain bankable and expandable adult-like microglia (NEL-MG) by using the head neuroepithelial layer in the mouse embryo (You et al., 2021).

## Conclusion

**FIGURE 3:**
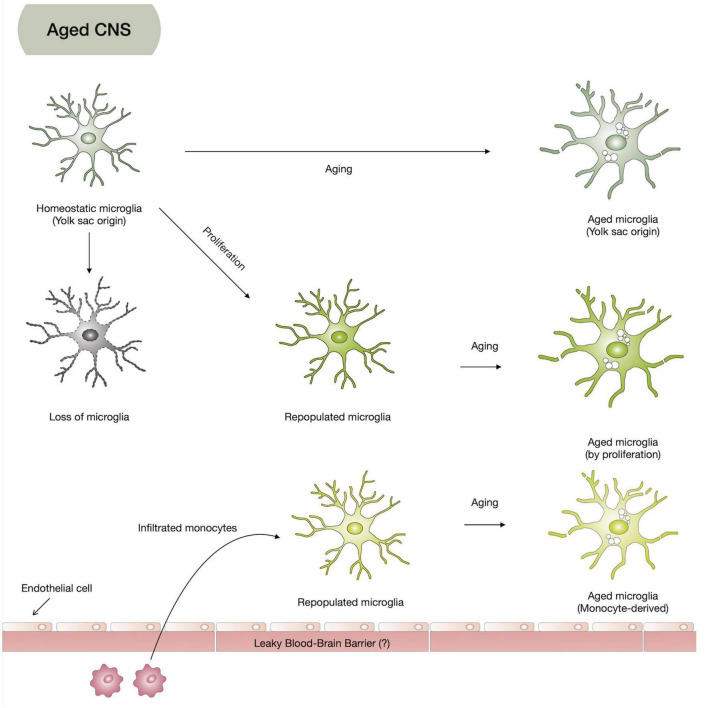
Hypothesis on aged microglia heterogeneity. Based on the microglia origin and their limited repopulation, aged microglia might be composed of yolk-sac-originated microglia (homeostatic microglia), repopulated microglia from infiltrated monocytes, and proliferation of homeostatic microglia.

Microglia are yolk-sac-derived CNS cells with distinct origins different from neurons, astrocytes, and oligodendrocytes. They are long-lived cells, and when they die by aging and other causes, they might be replaced by their proliferation or peripheral immune cells rather than being regenerated by their progenitors like other CNS cells, suggesting aged microglia heterogeneity (Figure 3). The function of microglia is also very extensive, affecting our brain homeostasis throughout life, from neurodevelopment to neurodegenerative changes. Due to the transcriptomic dissimilarity of the repopulating microglia and limited repopulating capacity, keeping the original resident microglia healthy for a long time seems to be another strategy to prevent neurodegenerative diseases. Moreover, with the evolving understanding of microglia, to understand the aging process of microglia, using further improved aged microglia models may provide us with a crucial key to find alternative therapeutic strategies for neurodegenerative diseases.

## Author Contributions

H-JY and M-SK wrote the manuscript. M-SK supervised all the processes, determined the direction of the manuscript, and approved the final submission of the manuscript. Both authors critically revised the manuscript and confirmed the author’s contribution statement.

## Conflict of Interest

The authors declare that the research was conducted in the absence of any commercial or financial relationships that could be construed as a potential conflict of interest.

## Publisher’s Note

All claims expressed in this article are solely those of the authors and do not necessarily represent those of their affiliated organizations, or those of the publisher, the editors and the reviewers. Any product that may be evaluated in this article, or claim that may be made by its manufacturer, is not guaranteed or endorsed by the publisher.
